# Classification of Comprehensive Neuro-Ophthalmologic Measures of Postacute Concussion

**DOI:** 10.1001/jamanetworkopen.2021.0599

**Published:** 2021-03-03

**Authors:** Christina N. Feller, May Goldenberg, Patrick D. Asselin, Kian Merchant-Borna, Beau Abar, Courtney Marie Cora Jones, Rebekah Mannix, Keisuke Kawata, Jeffrey J. Bazarian

**Affiliations:** 1 University of Rochester School of Medicine and Dentistry, Rochester, New York; 2 Medical College of Wisconsin, Milwaukee; 3 University of Rochester, Rochester, New York; 4 Department of Pediatrics, Boston Children’s Hospital, Harvard Medical School, Boston, Massachusetts; 5 Department of Emergency Medicine, University of Rochester School of Medicine and Dentistry, Rochester, New York; 6 Department of Kinesiology, Indiana University, Bloomington

## Abstract

**Importance:**

Symptom-based methods of concussion diagnosis in contact sports result in underdiagnosis and repeated head injury exposure, increasing the risk of long-term disability. Measures of neuro-ophthalmologic (NO) function have the potential to serve as objective aids, but their diagnostic utility is unknown.

**Objective:**

To identify NO measures that accurately differentiate athletes with and without concussion.

**Design, Setting, and Participants:**

This cohort study was conducted among athletes with and without concussion who were aged 17 to 22 years between 2016 and 2017. Eye movements and cognitive function were measured a median of 19 days after injury among patients who had an injury meeting the study definition of concussion while playing a sport (retrospectively selected from a concussion clinic), then compared with a control group of participants without concussion (enrolled from 104 noncontact collegiate athlete volunteers without prior head injury). Data analysis was conducted from November 2019 through May 2020.

**Exposure:**

Concussion.

**Main Outcomes and Measures:**

Classification accuracy of clinically important discriminator eye-tracking (ET) metrics. Participants’ eye movements were evaluated with a 12-minute ET procedure, yielding 42 metrics related to smooth pursuit eye movement (SPEM), saccades, dynamic visual acuity, and reaction time. Clinically important discriminator metrics were defined as those with significantly different group differences and area under the receiver operator characteristic curves (AUROCs) of at least 0.70.

**Results:**

A total of 34 participants with concussions (mean [SD] age, 19.7 [2.4] years; 20 [63%] men) and 54 participants without concussions (mean [SD] age, 20.8 [2.2] years; 31 [57%] men) completed the study. Six ET metrics (ie, simple reaction time, discriminate reaction time, discriminate visual reaction speed, choice visual reaction speed, and reaction time on 2 measures of dynamic visual acuity 2) were found to be clinically important; all were measures of reaction time, and none were related to SPEM. Combined, these 6 metrics had an AUROC of 0.90 (95% CI, 0.80-0.99), a sensitivity of 77.8%, and a specificity of 92.6%. The 6 metrics remained significant on sensitivity testing.

**Conclusions and Relevance:**

In this study, ET measures of slowed visual reaction time had high classification accuracy for concussion. Accurate, objective measures of NO function have the potential to improve concussion recognition and reduce the disability associated with underdiagnosis.

## Key Points

**Question:**

Which computerized eye-tracking metrics most accurately differentiate athletes with and without concussion after acute injury?

**Findings:**

In this cohort study of 34 college-age athletes with concussion and 54 uninjured athletes, 6 eye-tracking measures that reflect slowed visual reaction time had a combined sensitivity of 77.8% and specificity of 92.6% for discriminating between the groups.

**Meaning:**

These findings suggest that accurate, objective measures of slowed visual reaction time have the potential to improve concussion recognition and reduce the disability associated with underdiagnosis.

## Teaser

This cohort study identifies neuro-ophthalmologic measures that accurately differentiate athletes with and without concussion.

## Introduction

It is estimated that 1.6 to 3.8 million sports-related concussions occur annually in the United States.^1^ Accurate diagnosis is important for managing safe return to play (RTP) and academic activities. However, the current symptom-based method of concussion diagnosis has been criticized for being subjective,^2^ allowing injured athletes to downplay symptoms to avoid being removed from play.^3^ This can result in exposure to another head injury before recovery is complete, resulting in prolonged recovery.^4,5^ An objective diagnostic aid has the potential to improve concussion recognition and reduce or prevent disability associated with premature RTP.

Quantitative assessment of eye movements with eye tracking (ET) may be a potential solution. Concussion has been associated with abnormalities in all 4 basic eye movements, including saccades,^6,7,8^ smooth pursuit eye movement (SPEM),^9,10^ vergence movements,^11^ and vestibulo-ocular movements.^12^ These eye movements are executed by neural networks involving the midbrain, cerebellum, frontal eye fields, visual cortex, prefrontal cortex, and vestibular nuclear complex.^13^ Axonal injury in these networks is thought to underlie postconcussive imbalance, dizziness, and vision blurriness, especially when tracking objects in motion.^10,14^ Some eye movements (eg, predictive smooth pursuits) rely on intact working memory and attention to generate accurate prediction of future events, which is important for tracking moving objects.^13,15^ Thus, postconcussion impairments in cognitive processing may exacerbate eye movement performance.^16,17^ The associations between ET and cognitive processing are referred to as neuro-ophthalmologic (NO) function.^13^ Evaluation of NO response has been proposed as a way to detect neurologic injury after a head impact and aid in the diagnosis of concussion.^18,19,20^

Computerized ET can provide objective measures of SPEM (slow eye movements used to track a moving target) and saccades (rapid movements used to look from 1 target to another).^9,10,18,21,22,23,24,25,26,27,28,29,30,31,32,33^ SPEM metrics have been identified as accurate concussion classifiers,^29,34,35^ but the discriminatory accuracy of non-SPEMs and the contribution of attention and memory to eye movement performance have not, to our knowledge, been evaluated. Addressing these gaps would be crucial for identifying the ET metrics most likely to provide added value in the evaluation of concussion.

The objective of the current study was to identify ET metrics that most accurately distinguished athletes with and without concussion. Based on prior published work suggesting the potential clinical utility of SPEM, we hypothesized that reduced performance in these metrics would be the most accurate injury status classifiers. The secondary objective was to determine the contribution of attention and working memory to classifying ET metrics through correlation to validated measures of cognition. We hypothesized that reduced performance in classifying ET metrics after concussion would be associated with poorer visual memory or slower visual motor speed.

## Methods

### Participants and Setting

In this cohort study, eye movements and cognitive function were measured after the acute injury period (median, 19 days; range 2-457 days) in a group of athletes with concussion and compared with results from a control group without concussion. The postacute period was defined as more than 1 day after injury. Participants without concussion were accrued between January and March 2017 from a volunteer sample of 104 noncontact National Collegiate Athletic Association Division III athletes without a prior head injury history. Noncontact sports included tennis, golf, swimming, crew/rowing, and squash. Participants with concussion were retrospectively selected from 101 new patients presenting to a university sport concussion clinic for ongoing symptoms who underwent ET as part of a standardized clinical intake process between September 2016 and June 2017. To provide matching to the control group based on age and mechanism, participants aged 17 to 22 years injured playing a sport were selected from 101 patients with ET data. Study participants provided informed consent, and the research protocol was approved by the University of Rochester review board. This study followed the Strengthening the Reporting of Observational Studies in Epidemiology (STROBE) reporting guideline for cohort studies.

Concussion diagnostic criteria consisted of a head injury associated with either (1) witnessed or self-reported brief loss of consciousness, amnesia, or confusion or (2) onset within 24 hours of injury of headache, short-term memory concerns, dizziness, or imbalance.^36,37^ Concussion diagnosis was made by an experienced clinician using these criteria. Participants with traumatic intracranial lesions on head computed tomography scan, who were hospitalized for more than24 hours, or in whom an alternative diagnosis was suspected were excluded. All participants were excluded for history of strabismus. Participants requiring corrective lenses were not excluded but were required to wear them during the ET procedure. Observers were not masked to patient grouping.

### ET Procedure

Eye movements were evaluated with a commercially available eye tracker (RightEye) consisting of a 24-inch 3-dimensional vision monitor fitted with a System Management Interface 12-inch remote eye tracker (NVIDIA) connected to a gaming system (Alienware) and a wireless keyboard and mouse (Logitech).^38^ The eye tracker has a 120 Hz temporal resolution and an accuracy of 0.4°. The testing procedure lasted 12 minutes and produced 42 variables related to SPEM, saccades, dynamic visual acuity, and reaction time (eTable 1 in the Supplement). The testing parameters were factory preset and not modifiable. All participants underwent ET in a seated position after completing a 9-point calibration requiring fixation on at least 8 of 9 sequentially displayed points on the screen.

For circular smooth pursuits, participants tracked a black target of 0.2° diameter on a white background, which followed a circular trajectory of 10° radius at a rate of 25° per second (0.4 Hz) for 6.5 cycles (15 seconds). For horizontal smooth pursuits, participants tracked a dot (same size and speed as the circular smooth pursuit test) moving back and forth in a straight line horizontally across the screen for 25 seconds.^25^ To be on target, the participant was required to follow the stimulus within 2.4°. The protocol for vertical smooth pursuit was identical but in a vertical plane.^25^ The eye tracker measures reaction time in 3 dedicated tests (simple reaction time, choice reaction time, and discriminate reaction time) and as part of dynamic visual acuity testing.^39^ The eAppendix and eTable 2 in the Supplement include descriptions of these methods as well as those related to saccades.^39^

### Cognitive Testing

Cognitive performance was assessed using a software program (Immediate Post-Concussion Assessment and Cognitive Test [ImPACT Applications]) measuring total postconcussive symptom score and 4 domains of memory and attention (ie, verbal memory, visual memory, visual motor speed, and reaction time).^40^ Cognitive testing was administered immediately prior to ET in participants with concussion (eAppendix in the Supplement).

### Statistical Analysis

Important discriminator metrics were identified by comparing group differences in the 42 available metrics using the Mann-Whitney U test for variables with skew values greater than an absolute value of 2.0 and an unpaired *t* test for all others. Bonferonni correction was applied to the a priori 1-tailed *P* value indicating significance, moving the threshold from *P* < .05 to *P* < .0012. Area under the receiver operator characteristic curve (AUROC) was calculated for individual metrics in which there was a statistically significant difference between groups; those that also had an AUROC of at least 0.70 were considered clinically important.^41^ ET metrics that did not have an AUROC of at least 0.70 but nonetheless had moderate to large effect sizes (ie, Cohen *d*, >0.50) were considered potentially important. A multivariable logistic regression model was used to determine the AUROC combining all clinically important metrics and the Youden J index to determine the optimal probability cutoff for discriminating between groups. The Youden J index assigns equal weights to the misclassification of events by identifying the probability cutoff that maximizes the difference between true-positives and false-positives.^41^ This cutoff was used to determine the sensitivity and specificity of the model.

Because missing data were confined to the concussed group (eTable 1 in the Supplement), they were considered not missing at random and neither substituted nor imputed. Participants with missing data were included in the analysis, and each ET metric was analyzed with the available data. A sensitivity analysis using only participants with complete data was performed to estimate potential bias in results derived from participants with missing data.

Clinically important ET metrics were correlated with 4 cognitive domains and total symptom score in participants with concussions using the Spearman correlation coefficient. Similarly, the Spearman correlation coefficient was used to determine the association between the injury-ET time interval and values for each clinically important metric in participants with concussion. Statistical analyses were performed using SAS software version 9.4 (SAS Institute).

## Results

### Participant Characteristics

Thirty-four participants with concussion (mean [SD] age, 19.7 [2.4] years; 20 [63%] men) and 54 uninjured athletes (mean [SD] age, 20.8 [2.2] years; 31 [57%] men) completed the study. Of 101 available patients with concussion and ET data, 69 (69%) were either outside the age criteria or not injured during a sport, resulting in 32 (31%) participants with concussion. Of 104 healthy athlete volunteers, 50 (48%) were either ineligible or declined to participate, yielding 54 participants (52%) for the nonconcussed control group. Participants with concussion were most frequently injured playing soccer (6 [19%]), followed by football (7 [22%]) and basketball (4 [13%]) (Table 1). Compared with the control group, participants with concussion were younger and more likely to be White individuals (33 [61%] vs 28 [88%]). There was no significant group difference in the proportion of participants with attention-deficit/hyperactivity disorder. In participants with concussion, the median (interquartile range [IQR]) time between injury and ET procedure was 19 (10-39) days. Although the injury-testing interval ranged from 2 to 457 days, only 1 participant was tested after 58 days. Total symptom scores at the time of assessment ranged from 0 to 68 (median [IQR], 18 [4-39]). The 3 participants who reported a symptom score of 0 on cognitive testing had headache and/or dizziness with cognitive exertion. Because of ET software upgrades occurring during the course of study, a number of concussed participants (range, 1-13) were missing 1 or more reaction time variables (eTable 1 in the Supplement). In addition, cognitive test results were missing in 3 participants with concussion.

**Table 1.** Participant Characteristics

Characteristics	No. (%)	
	Athletes without concussion (n = 54)	Athletes with concussion (n = 32)
Age, mean (SD), y^a^	21 (2)	20 (2)
Men	31 (57)	20 (63)
Women	23 (43)	12 (37)
Race^a^		
White	33 (61)	28 (88)
Asian	13 (24)	2 (6)
African American	1 (2)	2 (6)
Hispanic	2 (4)	0
Other^b^	5 (9)	0
Handedness		
Right	48 (89)	27 (84)
Left	4 (7)	4 (13)
Both	2 (4)	1 (3)
ADD/ADHD	4 (7)	3 (9)
Sport^a^		
Squash	8 (15)	0
Tennis	14 (26)	0
Swim	8 (15)	0
Golf	5 (9)	0
Crew	19 (35)	0
Football	0	7 (22)
Cheerleading	0	2 (6)
Hockey	0	3 (9)
Lacrosse	0	1 (3)
Basketball	0	4 (13)
Soccer	0	6 (19)
Field hockey	0	2 (6)
Wrestling	0	1 (3)
Ski	0	1 (3)
Other^c^	0	5 (16)

Abbreviations: ADD, attention-deficit disorder; ADHD, attention-deficit/hyperactivity disorder.

^a^ *P* < .05.

^b^ Other race includes American Indian or Alaska Native, Native Hawaiian or other Pacific Islander.

^c^ Other sports include (1 participant for each) rugby, equestrian, sailing, snowboarding, and motocross.

### ET Metrics in Participants With and Without Concussion

**Figure 1.**
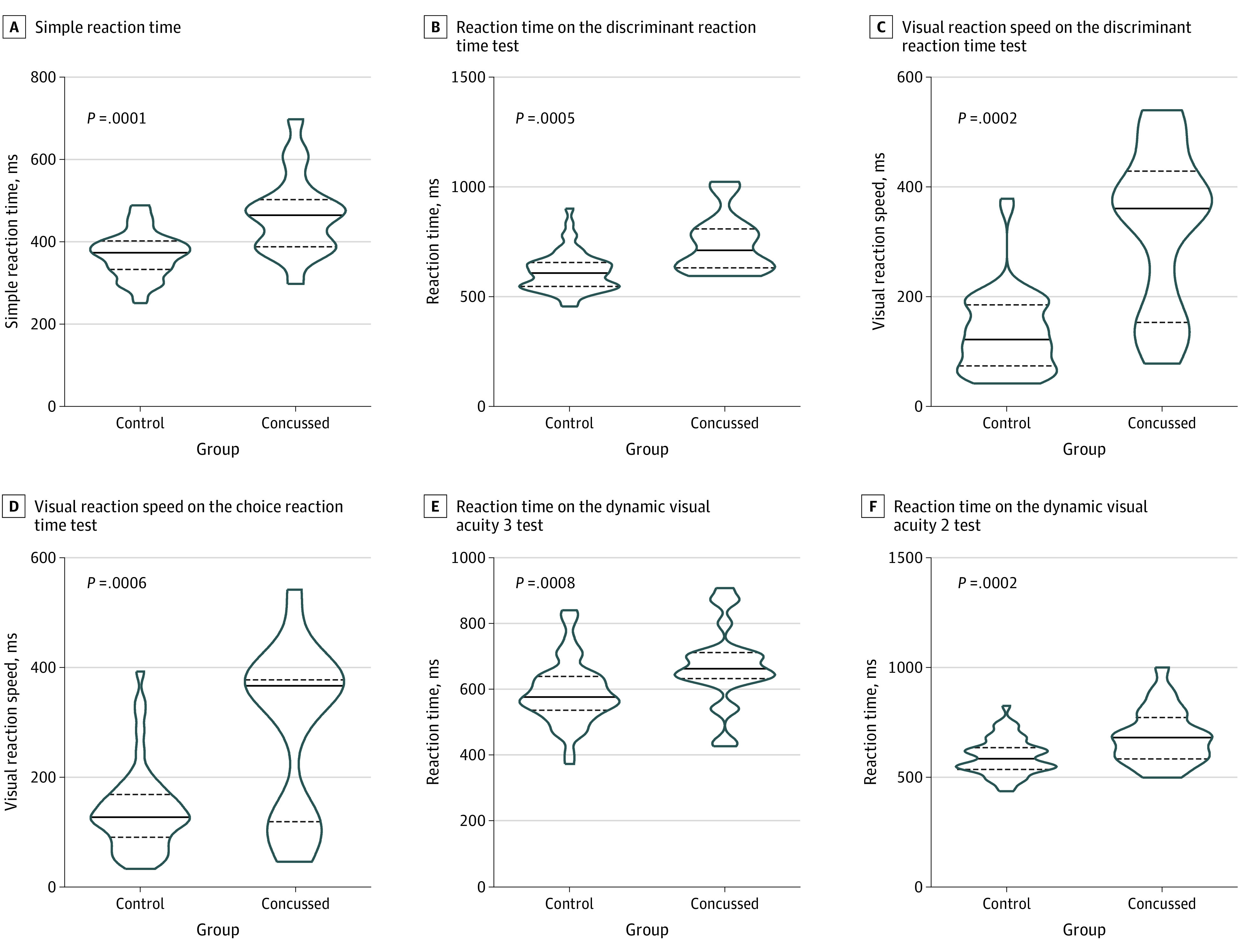
Comparison of Clinically Important Eye-Tracking Metrics in Concussed and Control Athletes Truncated violin plots displaying the rotated kernel density for each of the 6 clinically important eye tracking metrics in participants with and without concussion, compared using unpaired *t* test with a Bonferonni-corrected *P* < .0012. Solid line indicates median; dashed lines, interquartile range.

The distributions of 8 of the 42 ET metrics (19%) were significantly different between participants with and without concussion after Bonferonni correction. Of these 8 ET metrics, 6 (75%) had an AUROC of at least 0.70 and were thus considered clinically important; all were measures of reaction time and none were related to SPEM (Table 2). They were simple reaction time, discriminate reaction time, discriminate visual reaction speed, choice visual reaction speed, and reaction time on 2 measures of dynamic visual acuity. Discriminatory accuracy ranged from an AUROC of 0.72 (95% CI, 0.61-0.84) for the reaction time metric in the dynamic visual acuity 2 test to an AUROC of 0.81 (95% CI, 0.70-0.93) for simple reaction time. All 6 clinically important measures of reaction time were slower among participants with concussion (Figure 1). For example, median (IQR) simple reaction time among participants with concussion was significantly slower than among participants without concussion (464.9 [387.9-502.7] milliseconds vs 373.5 [332.9-402.3] milliseconds). Despite the broad range in postinjury test assessment, none of these measures correlated with the injury-ET time interval (eTable 3 in the Supplement). The combination of these 6 clinically important metrics had an AUROC of 0.90 (95% CI, 0.80, 0.99) (Table 2). The Youden J Index identified 0.37 as an optimal probability cutoff, which yielded a sensitivity of 77.8% and specificity of 92.6%.

**Table 2.** Classification Accuracy of Clinically Important Eye Tracking Metrics

Metric	AUROC (95% CI)	*P* value
Simple reaction time; reaction time, ms	0.81 (0.70-0.93)	.0001
Discriminate reaction time, ms		
Reaction time	0.80 (0.70-0.91)	.0005
Visual reaction speed	0.80 (0.66-0.94)	.0002
Choice reaction time; visual reaction speed, ms	0.76 (0.60-0.91)	.0006
Dynamic visual acuity 3; reaction time, ms	0.74 (0.62-0.86)	.0008
Dynamic visual acuity 2; reaction time, ms	0.72 (0.61-0.84)	.0002
All 6 metrics combined^a^	0.90 (0.80-0.99)	<.0001

Abbreviation: AUROC, area under receiver operator characteristic curve.

^a^ AUROC determined using multivariable logistic regression.

A sensitivity analysis of participants with complete data for all ET metrics showed no change in statistical significance of the AUROC for the 6 clinically important metrics. There were small changes in AUROC point estimates that ranged from −0.02 to 0.05 (eg, choice reaction time, visual reaction speed, main analysis: AUROC, 0.76; 95% CI, 0.60-07.91; sensitivity analysis: AUROC, 0.74; 95% CI, 0.48-0.90; difference, −0.02; dynamic visual acuity 3, reaction time, main analysis: AUROC, 0.74; 95% CI, 0.62-0.86; sensitivity analysis: AUROC, 0.79; 95% CI, 0.58-0.90; difference, 0.05).

**Figure 2.**
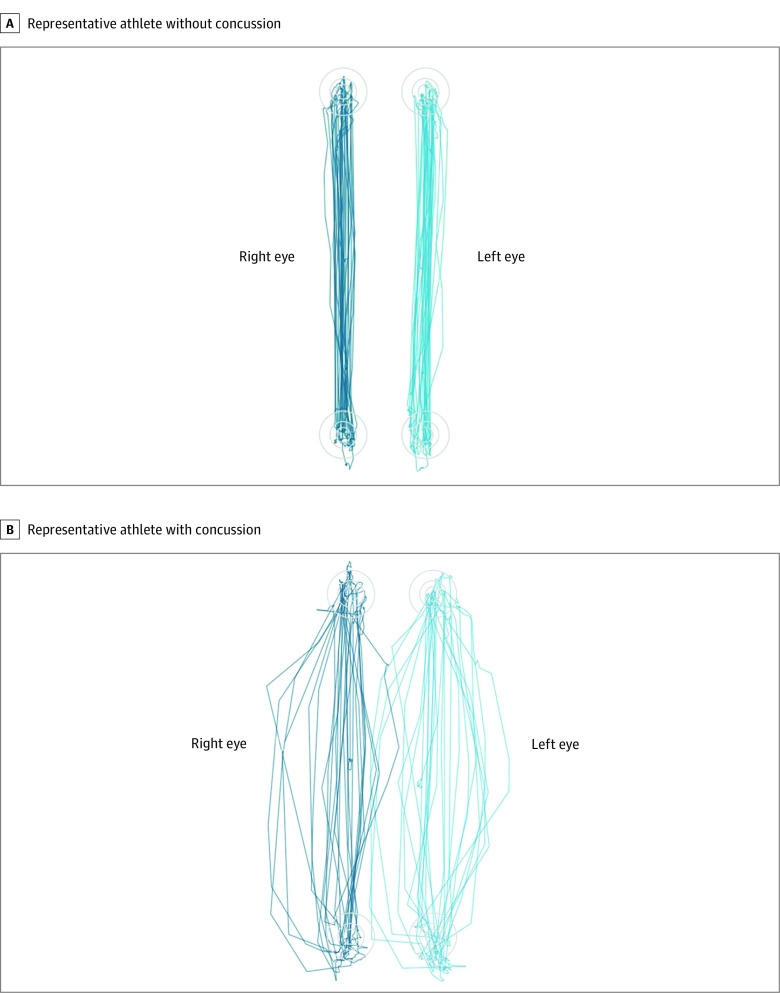
Vertical Saccades in a Representative Athlete With Concussion and Control Athlete Although not meeting criteria as clinically important, 6 metrics related to vertical saccades had moderate discriminatory effect sizes (Cohen *d*, >0.50). The increased number of target misses can be seen in a representative athlete with concussion (B) compared with a representative control athlete (A). The saccadic pathways are also more irregular and less linear in the athlete with concussion, although this eye movement feature is not measured by the eye tracker.

Although 2 of the 8 variables (25%) with significantly different group means did not have an AUROC of at least 0.70, they nonetheless had large effect sizes (Cohen *d*, >0.80). An additional 8 variables with statistically nonsignificant group differences had moderate effect sizes (Cohen *d*, >0.50); 6 (75%) of these involved vertical saccadic eye movements (Figure 2). These 10 potentially important variables are summarized in eTable 4 in the Supplement.

### Correlation of ET Variables With Currently Used Cognitive Performance Measures

Among participants with concussion, 3 of the 6 clinically important ET metrics (50%) had significant correlations to cognitive test measures of reaction time (Table 3). Simple reaction time also correlated with total symptom score. The remaining 3 clinically important ET metrics, including 2 measuring visual reaction speed, were not correlated with cognitive test–measured reaction time. For all 6 metrics, correlations to verbal memory, visual memory, and visual motor speed scores were weak and statistically nonsignificant.

**Table 3.** Correlation Between Clinically Significant Eye-Tracking Metrics and Measures of Cognitive Performance in Participants with Concussion

Eye-tracking metric	Participants with concussion, No.	Correlation coefficient				
		Memory		Visual motor speed	Reaction time	Total symptom
		Verbal	Visual			
Simple reaction time; reaction time, ms	22	−0.243	−0.421	−0.202	0.425^a^	0.628^b^
Discriminate reaction time, ms						
Reaction time	17	0.116	−0.101	−0.287	0.358	0.298
Visual reaction speed	17	0.386	0.275	0.083	−0.186	0.152
Choice reaction time; visual reaction speed, ms	17	0.107	0.098	−0.200	−0.026	0.395
Dynamic visual acuity 3; reaction time, ms	25	−0.137	−0.320	−0.280	0.623^b^	−0.036
Dynamic visual acuity 2; reaction time, ms	27	−0.281	−0.347	−0.211	0.415^a^	0.033

^a^ *P* < .05.

^b^ *P* < .01.

## Discussion

Contrary to our primary hypothesis, ET measures of reaction time, not SPEM, were the most accurate classifiers of athletes with and without concussion. Of the 6 most discriminatory, clinically important ET measures, all involved reaction time. Participants with concussion had significantly slower reaction time and impaired ability to see a fast-moving target during a dynamic visual acuity task compared with a control group without concussion. Participants with concussion also had slower reaction time during simple and discriminate reaction time tests as well as slower visual reaction speed during choice and discriminate reaction time tests. Moreover, slower simple reaction time was associated with higher total post concussive symptom scores. Individually, the AUROC for these 6 ET metrics ranged from 0.72 to 0.81, while their combined AUROC was 0.90.

Our results indicate that concussion poses a significant challenge to responding to a rapidly appearing visual target and that the components of visual reaction time across multiple tests may offer an objective, clinical tool to diagnose concussion after the acute postinjury phase. This information may be especially useful for office-based health care professionals who evaluate patients with concussion days to weeks after injury. Pending confirmation in an independent cohort, visual reaction time measures could be used as a diagnostic aid when symptom minimization is suspected or when the clinical picture is otherwise unclear.

Although others have reported postconcussive slowing in visual reaction time (often simply called reaction time),^42^ our finding of slowed visual reaction speed is novel. As the participant fixes on a central target, visual reaction time measures the time elapsed between presentation of a peripheral target and a manual motor response, such as pressing a mouse button or computer key. However, this single measure involves 3 separate processes: saccadic latency, visual reaction speed, and processing speed. Visual reaction speed (also called saccadic velocity) measures the time required for the eyes to travel from the central target to the peripheral target. Unlike overall visual reaction time, visual reaction speed does not involve a manual motor response and can only be measured by a device that monitors eye movements, such as an eye tracker. In our study, the finding that neither ET measure of visual reaction speed correlated with reaction time on cognitive testing suggests that this metric may be measuring aspects of visual reaction time unrelated to cognitive processes and more specific to disruption in oculomotor pathways, potentially providing unique clinical information for identifying concussion and determining the optimal timing of RTP. While slowed visual reaction speed after concussion has been reported by at least 1 other author,^26^ anatomic associations were not investigated nor was its value as a classifier or outcome predictor.

Contrary to our secondary hypothesis, performance on the 6 clinically important classifying metrics was not correlated with visual memory or visual motor speed. There are several potential explanations for this. First, our secondary hypothesis was linked to the underlying assumption that metrics related to SPEM would be the most important injury classifies, not reaction time measures. Prior research indicated that variation in performance of SPEM and saccadic eye movements was associated with variation in cognitive processes, such as working memory and visual motor speed.^43,44^ Based on our results, these cognitive processes may have less of an influence on visual reaction time measures. Furthermore, our inability to identify a significant correlation to cognitive processes may be owing to relatively small sample size compounded by missing visual reaction time and/or cognitive test measures in several participants. However, the finding that visual reaction time variables on ET correlated fairly robustly to reaction time scores on cognitive testing suggests that the sample size was adequate.

In addition to identifying 6 clinically important ET metrics, we identified an additional 10 potentially important metrics, most (6 of 10) related to vertical saccades. Participants with concussion displayed more target misses, fewer fixations, and fewer overall saccadic eye movements compared with their counterparts without concussion. Several authors have reported significant postconcussive deficits in vertical saccades.^23,25,30,45^ It is unclear why vertical saccade metrics were not statistically significant classifiers in our study. Small sample size combined with the relatively conservative Bonferonni method of correcting for multiple comparisons may have obscured a statistically significant group difference.

While promising, additional research is needed to confirm our findings in an independent cohort and to determine the extent to which visual reaction time metrics improve at the time of RTP. Persistence of slowed visual reaction time after RTP could offer biologic insight into decrements in athletic performance and increases in injuries observed after concussed athletes RTP.^46,47,48,49^ In addition, future studies on the ability of ET metrics to predict prolonged (ie, >3 months) concussion recovery would provide clinicians and researchers with a useful risk stratification tool. Finally, research to identify structural correlates of slowed visual reaction time using advanced neuroimaging, such as diffusion tensor imaging, would be important for confirming a causal relationship to concussion.

### Limitations

This study has several limitations. Unlike participants in the control group, those with concussion may have been exposed to repetitive head impacts (RHIs) which can affect oculomotor function and bias our results away from the null. Although RHIs have been associated with acute and transient worsening in near-point convergence,^50,51^ they have not been associated with significant changes in ET metrics.^52^ Athletes in the control group who played sports requiring advanced hand-eye coordination skills, such as tennis and squash, may have had better baseline visual reaction time than athletes with concussion who primarily played football, potentially accentuating group differences in ET measures. The low temporal resolution of the ET device (120 Hz) compared with laboratory-based research devices may have prevented detection of small reaction time differences. However, the temporal sampling error for a 120 Hz device is estimated to be approximately 4 milliseconds,^53^ well below the observed group differences in reaction time measures, which ranged from 23.277 to 185.018 milliseconds (eTable 1 in the Supplement). Moreover, the reliability of reaction time measures on the device used in our study was quite robust among noninjured young adults, with most intraclass correlation coefficients above 0.75.^54^ Because visual reaction time and other eye movement performance metrics are faster and more accurate in athletes than nonathletes^23^ and decline with advancing age,^54,55,56^ our results may not be generalizable to older or nonathlete populations. Furthermore, although observers were not masked to patient grouping, neither ET nor cognitive testing are subjective measures, likely limiting the effect of observer bias.

## Conclusions

Contrary to our primary hypothesis, our results indicated that ET measures of visual reaction time, but not SPEM, were the best NO classifiers of athletes with and without concussion. Six ET measures reflecting slowed visual reaction time had a sensitivity of 77.8% and specificity of 92.6% for discriminating between the groups. None of these 6 metrics correlated with visual memory or visual motor speed, and 3 of the 6 did not correlate to reaction time on cognitive testing. Thus, not only were these metrics highly discriminatory, they may be measuring aspects of visual reaction time unrelated to cognitive processes and more specific to disruption in oculomotor pathways, potentially providing unique clinical information for identifying and managing a concussion. Providing objective measurements of visual reaction time may improve concussion diagnosis and management and reduce the disability associated with premature return to play.

## Notes

Supplement. **eAppendix. **Description of Eye-Tracking Tests of Saccades and Description of Cognitive Testing **eTable 1. **Comparison of All Eye-Tracking Metrics in Participants With and Without Concussion **eTable 2. **Description of Eye-Tracking Tests of Reaction Time and Dynamic Visual Acuity **eTable 3. **Correlation Between Clinically Important Eye-Tracking Metrics and Injury–Eye Tracking Time Interval **eTable 4. **Classification Accuracy of Potentially Important Eye Tracking
