# Error in Byline

**DOI:** 10.1001/jamanetworkopen.2021.7894

**Published:** 2021-03-31

In the Original Investigation “Classification of Comprehensive Neuro-Ophthalmologic Measures of Postacute Concussion,”^1^ published March 3, 2021, there was an error in the byline. Christina Feller’s degrees should have appeared as BA, BS. This article has been corrected.^1^
