# Supplementary material for: Classification of Comprehensive Neuro-Ophthalmologic Measures of Postacute Concussion
Source: JAMA Netw Open. 2021 Mar 3;4(3):e210599. doi: 10.1001/jamanetworkopen.2021.0599 (PMC7930925; doi:10.1001/jamanetworkopen.2021.0599)
Supplement: Supplement. — eAppendix. Description of Eye-Tracking Tests of Saccades and Description of Cognitive Testing eTable 1. Comparison of All Eye-Tracking Metrics in Participants With and Without Concussion eTable 2. Description of Eye-Tracking Tests of Reaction Time and Dynamic Visual Acuity eTable 3. Correlation Between Clinically Important Eye-Tracking Metrics and Injury–Eye Tracking Time Interval eTable 4. Classification Accuracy of Potentially Important Eye Tracking [file jamanetwopen-e210599-s001.pdf]

Supplemental Online Content

Feller CN, Goldenberg M, Asselin PD, et al. Classification of comprehensive neuro-ophthalmologic measures of postacute concussion. *JAMA Netw Open*. 2021;4(3):e210599. doi:10.1001/jamanetworkopen.2021.0599

eAppendix. Description of Eye-Tracking Tests of Saccades and Description of Cognitive Testing
eTable 1. Comparison of All Eye-Tracking Metrics in Participants With and Without Concussion
eTable 2. Description of Eye-Tracking Tests of Reaction Time and Dynamic Visual Acuity
eTable 3. Correlation Between Clinically Important Eye-Tracking Metrics and Injury–Eye Tracking Time Interval
eTable 4. Classification Accuracy of Potentially Important Eye Tracking

This supplemental material has been provided by the authors to give readers additional information about their work.

© 2021 Feller CN et al. *JAMA Network Open.*

eAppendix. Description of Eye-Tracking Tests of Saccades and Description of Cognitive Testing

Description of eye tracking tests of saccades

In the horizontal saccade test, subjects were required to focus on a target in the center of the screen before moving their eyes back and forth for 10 seconds between two peripherally appearing, 1 cm diameter white dots on a black background, 10 cm apart. Their goal was to 'target each dot' on the left and right of the screen as quickly and accurately as possible. The same procedure was performed in a vertical plan for the vertical saccade test.

Description of Cognitive testing

This test is performed with the subject responding to visual cues on a computer screen by clicking a mouse or specific keyboard keys. Eye movements are not measured. Unlike eye-tracking, cognitive testing measures reaction time by combining results from 3 separate subtests. X's & O's (choice reaction time), Symbol Match (choice reaction time with a memory and learning task), and Color Match (choice and discriminate reaction time).

© 2021 Feller CN et al. *JAMA Network Open.*

eTable 1. Comparison of All Eye-Tracking Metrics in Participants With and Without Concussion

Metric[a]	Missing data[b]	t	t-test for Equality of Means						
			df	p-value[c]	Mean Difference	Std. Error Difference	95% CI of the Difference		Cohen's d
							Lower	Upper	
**SMOOTH PURSUITS**									
Circular									
Smooth Pursuit (%)	0/0	1.924[f]	41.784	0.101[g]	4.747	2.467	-0.232	9.726	0.50
Saccade(s) (%)	0/0	-1.880[f]	42.225	0.092[g]	-4.495	2.391	-9.320	0.330	0.48
Eye/Target Velocity Error (°/s)	0/0	-0.453	84	0.652	-0.199	0.441	-1.077	0.678	0.10
Horizontal synchronization (0-1)	0/0	1.459	84	0.148	0.013	0.009	-0.005	0.031	0.33
Vertical Synchronization (0-1)	0/0	0.954	84	0.343	0.013	0.013	-0.014	0.038	0.21
Predictive Smooth Pursuit (%)	0/0	0.667	84	0.921[g]	1.261	1.891	-2.499	5.020	0.15
Smooth Pursuit On Target (%)	0/0	1.430	84	0.156	6.043	4.227	-2.362	14.448	0.32
Latent Smooth Pursuit (%)	0/0	-0.857	84	0.394	-2.530	2.950	-8.397	3.337	0.19
Horizontal									
Eye/Target Velocity Error (°/s)	0/0	1.572	84	0.120	1.203	0.766	-0.319	2.726	0.35
Horizontal synchronization (0-1)	0/0	-1.382	84	0.181[g]	-0.006	0.005	-0.015	0.003	0.31
Smooth Pursuit (%)	0/0	-0.173	84	0.975[g]	-0.423	2.442	-5.279	4.434	0.04
Saccade(s) (%)	0/0	-1.338[f]	43.835	0.348[g]	-1.573	1.175	-3.942	0.796	0.34
Vertical									
Eye/Target Velocity Error (°/s)	0/0	-0.473	83	0.638	-0.362	0.765	-1.884	1.160	0.11
Horizontal synchronization (0-1)	0/0	0.050	83	0.960	0.001	0.014	-0.027	0.028	0.01
Smooth Pursuit (%)	0/0	-1.566[f]	49.546	0.124	-2.820	1.801	-6.437	0.798	0.06
**SACCADES**									
Horizontal									
Saccade(s) (#), R	0/0	1.742	84	0.085	2.865	1.645	-0.406	6.136	0.39
Saccade(s) (#), L	0/0	1.781	84	0.079	2.927	1.644	-0.342	6.196	0.40
Fixations (#), R	0/0	1.742	84	0.085	2.865	1.645	-0.406	6.136	0.39
Fixations (#), L	0/0	1.781	84	0.079	2.927	1.644	-0.342	6.196	0.40
Target Misses (#), R	0/0	1.770	84	0.080	3.089	1.746	-0.382	6.561	0.39

© 2021 Feller CN et al. *JAMA Network Open.*

Target Misses (#), L	0/0	1.946	84	0.006[g]	3.058	1.572	-0.068	6.183	0.43
Vertical									
Saccade(s) (#), R[e]	0/0	2.700	84	0.008	3.579	1.326	0.942	6.215	0.60
Saccade(s) (#), L[e]	0/0	2.709	84	0.008	3.597	1.328	0.956	6.238	0.60
Fixations (#), R[e]	0/0	2.700	84	0.008	3.579	1.326	0.942	6.215	0.60
Fixations (#), L[e]	0/0	2.709	84	0.008	3.597	1.328	0.956	6.238	0.60
Target Misses (#), R[e]	0/0	2.900	84	0.005	3.773	1.301	1.186	6.360	0.65
Target Misses (#), L[e]	0/0	2.796	84	0.006	3.469	1.241	1.002	5.936	0.62
**DYNAMIC VISUAL ACUITY**									
Head moving, object still									
Acuity (20/x)	0/0	0.196	82	0.845	1.576	8.049	-14.436	17.589	0.04
Head still, object moving									
Speed (mph)[d]	1/0	-5.381[f]	31.441	<0.001	-34.815	6.470	-48.003	-21.626	1.57
Reaction time (ms)[d]	2/0	-3.915	82	<0.001	-86.767	22.160	-130.850	-42.683	0.89
Head and object moving									
Speed (mph)[d]	1/0	-5.486[f]	31.442	<0.001	-34.511	6.291	-47.334	-21.687	1.60
Reaction time (ms)[d]	4/0	-3.482	80	0.001	-81.672	23.458	-128.354	-34.990	0.81
**REACTION TIME**									
Simple									
Reaction Time (ms)[d]	8/0	-4.518[f]	29.928	<0.001	-92.500	20.475	-134.320	-50.680	1.35
Choice									
Visual Reaction Speed (ms)[d]	13/0	-4.034[f]	21.416	0.001	-142.248	35.264	-215.495	-68.999	1.44
Processing Speed (ms)	13/0	-1.027	71	0.308	-23.227	22.616	-68.322	21.868	0.27
Reaction Time (ms)[e]	13/0	-2.421	71	0.007[g]	-82.201	33.951	-149.898	-14.504	0.65
Response accuracy (%)	8/0	-1.116	76	0.268	-3.338	2.992	-9.297	2.621	0.27
Discriminate									
Visual Reaction Speed (ms)[d]	13/0	-4.566[f]	20.478	<0.001	-185.018	40.523	-269.421	-100.614	1.72
Processing Speed (ms)[e]	13/0	-2.096	71	0.040	-46.849	22.348	-91.409	-2.288	0.56
Reaction Time (ms)[d]	13/0	-4.034[f]	24.342	0.001	-126.614	31.387	-191.346	-61.882	1.28
Response accuracy (%)	8/0	-1.479	76	0.143	-2.162	1.462	-5.074	0.750	0.36

© 2021 Feller CN et al. *JAMA Network Open.*

[a]All metrics are binocular unless otherwise indicated. [b]N: number of concussed subjects / number of non-concussed controls with missing data. [c]42 total comparisons means the Bonferonni corrected p value is < 0.0012. [d]Clinically important metrics (see text for definition). [e]Metrics that did not qualify as clinically important but had moderate-high effect sizes. [f]Levene's test indicates unequal group variances ($p < 0.05$), such that Welch-Satterthwaite adjustments to df were required. [g]Distribution was skewed (>|2.0|), such that a Mann-Whitney U test was in place of an independent measures *t*-test. Mean difference data provided for consistency of presentation. ms: milliseconds. mph: miles per hour.

© 2021 Feller CN et al. *JAMA Network Open.*

eTable 2. Description of Eye-Tracking Tests of Reaction Time and Dynamic Visual Acuity

Test	Description	Output Metrics
**Simple Reaction Time**	Subject looks at a 3 cm target in the shape of the solar system in the center of the screen. When eyes are confirmed to be looking at the target, it changes shape randomly. Subject must press number 1 on keyboard when the target changes to an alien symbol. Results are an average across 8 trials. Two practice trials were given before the eight test trials.	**Reaction Time** (time in milliseconds from appearance of alien symbol to keypress)
**Discriminate Reaction Time**	Subject looks at a 3 cm target in the shape of the solar system in the center of the screen. When eyes are confirmed to be looking at the target, an arrow moved out from the center in one of four directions (up, down, left, or right) for 8 cm. At the end of the arrow, a stimulus was presented. There were three stimulus choices. Only one stimulus required a response from the participant, which was to press the number 1 button on the keyboard. Results are an average across 8 trials. Four practice trials were given before the 8 test trials.	**Saccadic Latency** (elapsed time in milliseconds from appearance of a peripheral stimulus to eyes moving off the central target) **Visual Reaction Speed** (time in milliseconds from eyes moving off the central target to hitting the peripheral stimulus) **Processing Speed** (time in milliseconds from eyes hitting the peripheral stimulus to keypress) **Reaction Time** (sum total time in milliseconds of SL+VRS+PS)
**Choice Reaction Time**	Subject looks at a 3 cm target in the shape of the solar system in the center of the screen. When eyes are confirmed to be looking at the target, an arrow moved out from the center in one of four directions (up, down, left, or right) for 8 cm. A stimulus was presented at the end of the arrow once the final location was reached. There were three stimulus choices, each requiring a different response. There was one response per stimulus (e.g., number 1 button, number 2 button). Results are an average across 8 trials. Four practice trials were given before the 8 test trials.	Same as above
**Dynamic Visual Acuity 1:** Head moving, object still	Using a 200 metronome sound at a rate of 2-hertz, subjects rotate their head left and right while keeping their eyes focused on a black box in the center of the screen. At random 0-2 sec intervals, a Landolt-C of constant 20-100 size but varying orientation (up, down, left, right) appears in the center of the screen. The participant is given up to 2 seconds to press the keyboard arrow key that corresponds to the Landolt-C orientation.	**Acuity** (20/x)
**Dynamic Visual Acuity 2:** Head still, object moving	The subjects head is fixed. At random starting intervals, a Landolt-C moves across the screen from left to right at speeds ranging from 3 to 63 mph. The size of the Landolt-C is a constant 20-100 size but the orientation varies: up, down, left, right. The participant	**Speed of the stimulus** (mph) **Reaction Time** (time in milliseconds to keypress)

© 2021 Feller CN et al. *JAMA Network Open.*

	is given up to 2 seconds to press the keyboard arrow key that corresponds to the Landolt-C orientation.	
**Dynamic Visual Acuity 3:** Head and object moving	As above, but the subjects head is allowed to move freely.	**Speed of the stimulus** (mph)<br>**Reaction Time** (time in milliseconds to keypress)

© 2021 Feller CN et al. *JAMA Network Open.*

eTable 3. Correlation Between Clinically Important Eye-Tracking Metrics and Injury–Eye Tracking Time Interval

**Eye Tracking Metric**	**N**	**Injury-eye Tracking Time Interval**	
		***r*-value**[a]	**p-value**
Simple Reaction Time: Reaction Time (ms)	21	-0.140	0.5492
Discriminate Reaction Time: Reaction Time (ms)	19	-0.054	0.8303
Discriminate Reaction Time: Visual Reaction Speed (ms)	19	-0.161	0.5148
Choice Reaction Time: Visual Reaction Speed (ms)	19	-0.275	0.2594
Dynamic Visual Acuity 3: Reaction Time (ms)	26	-0.217	0.2900
Dynamic Visual Acuity 2: Reaction Time (ms)	27	-0.303	0.1252

N: number of subjects. ms: milliseconds. [a]Spearman's correlation coefficient

© 2021 Feller CN et al. *JAMA Network Open.*

**eTable 4. Classification Accuracy of Potentially Important Eye Tracking**			
**Metric**	**p-value**	**AUROC**	**Cohens D**
Dynamic Visual Acuity 3: Speed (mph)	<0.0001	0.70	1.60
Dynamic Visual Acuity 2: Speed (mph)	<0.0001	0.68	1.57
Vertical Saccades, Target Misses (#), R	0.0026	NC	0.65
Choice Reaction Time: Reaction Time (ms)	0.0047	NC	0.65
Vertical Saccades, Target Misses (#), L	0.0041	NC	0.62
Vertical Saccades, Saccades (#), R	0.0084	NC	0.60
Vertical Saccades, Saccades (#), L	0.0078	NC	0.60
Vertical Saccades, Fixations (#), R	0.0081	NC	0.60
Vertical Saccades, Fixations (#), L	0.0078	NC	0.60
Discriminate Reaction Time: Processing Speed (ms)	0.040	NC	0.56

NC: not calculated because p-value $\geq$0.0012. mph: miles per hour. ms: milliseconds.

© 2021 Feller CN et al. *JAMA Network Open.*
